# Performance targets defined by retro-techno-economic analysis for the use of soybean protein as saccharification additive in an integrated biorefinery

**DOI:** 10.1038/s41598-020-64316-6

**Published:** 2020-04-30

**Authors:** Mariana G. Brondi, Andrew M. Elias, Felipe F. Furlan, Roberto C. Giordano, Cristiane S. Farinas

**Affiliations:** 1 Embrapa Instrumentation, Rua XV de Novembro 1452, 13560-970 São Carlos, SP Brazil; 2 Graduate Program of Chemical Engineering, Federal University of São Carlos, 13565-905 Sao Carlos, SP Brazil

**Keywords:** Biotechnology, Energy science and technology, Engineering

## Abstract

The use of additives in the enzymatic saccharification of lignocellulosic biomass can have positive effects, decreasing the unproductive adsorption of cellulases on lignin and reducing the loss of enzyme activity. Soybean protein stands out as a potential lignin-blocking additive, but the economic impact of its use has not previously been investigated. Here, a systematic evaluation was performed of the process conditions, together with a techno-economic analysis, for the use of soybean protein in the saccharification of hydrothermally pretreated sugarcane bagasse in the context of an integrated 1G-2G ethanol biorefinery. Statistical experimental design methodology was firstly applied as a tool to select the process variable solids loading at 15% (w/w) and soybean protein concentration at 12% (w/w), followed by determination of enzyme dosage at 10 FPU/g and hydrolysis time of 24 h. The saccharification of sugarcane bagasse under these conditions enabled an increase of 26% in the amount of glucose released, compared to the control without additive. The retro-techno-economic analysis (RTEA) technique showed that to make the biorefinery economically feasible, some performance targets should be reached experimentally such as increasing biomass conversion to ideally 80% and reducing enzyme loading to 5.6 FPU/g in the presence of low-cost soybean protein.

## Introduction

In order to reduce the carbon footprint of fossil fuel consumption, the bioconversion of lignocellulosic biomass into biofuels such as cellulosic (2G) ethanol offers an attractive sustainable alternative that complies with the concepts of biorefinery and bio-economy^1–3^. However, the production cost of 2G ethanol is still high, due to the existence of technological bottlenecks such as the low yield of the hydrolysis reaction and the high cost of the cellulolytic enzymes^4–6^. The nonproductive adsorption of cellulases on lignin leads to lower saccharification yields and the need for higher enzyme loadings^7–9^. In order to minimize this negative effect of lignin, the use of additives to mitigate the nonproductive binding has been investigated^10–13^. Among the additives that can be used to reduce the nonproductive adsorption of enzymes are non-ionic surfactants (Tween)^14,15^, polymers (PEG - polyethylene glycol)^16,17^ and noncatalytic proteins (BSA - bovine serum albumin)^11,13,16^, that bind into lignin mostly through hydrophobic interaction^18,19^. However, there is still a need to evaluate the techno-economic impacts of these additives in the context of biorefineries for the production of 2G ethanol in large-scale industrial processes. Also, the additives must be low-cost in order to make this approach economically feasible.

Soybean protein stands out as a potential lignin-blocking additive to be used in biorefineries due to its low cost and the global abundance of soybean^5,20^. Recent studies of the use of soybean protein during the saccharification of different lignocellulosic biomasses including sugarcane bagasse, bamboo, and wood chips resulted in up to 76% improvement in the amount of glucose released^11,21–23^. The addition of soybean protein during the hydrolysis of sugarcane bagasse enabled the enzyme dosage to be reduced by 50%, while maintaining the same glucose release efficiency^11,21^. Furthermore, when compared to the saccharification yield achieved with the addition of BSA, soybean protein had a greater effect in enhancing the reaction yield, with these positive results being attributed to the mitigation of nonproductive adsorption^11,21,23^. Given these positive findings concerning the use of soybean protein to improve the saccharification yield, it is crucial to evaluate the techno-economic impact of its addition in the 2G ethanol production process, in the biorefinery context.

The techno-economic analysis of the 2G ethanol production has been recently addressed by the literature, for different feedstocks and process configurations. For instance, correlations between biomass composition and the economic impact on bioethanol production have been proposed^24^. Other studies have described the role of lignin as a source for high-valued co-products on the economics of an integrated biorefinery^25^. Economic and technological projections until 2030 have also been used to assess 1G and 2G ethanol production costs^26^. In addition, the impact of shifting between electric energy and 2G ethanol production has been evaluated^27^. A key point shown is that the association of 2G ethanol production process with 1G ethanol is an important way to ensure economic feasibility^26–29^. However, there is still a need to provide targets to be pursued by the R&D teams to improve the performance of the technology in order to make the integrated biorefinery economically feasible.

One innovative way of performing process economic evaluation, developed by Furlan *et al*.^30^ and applied by Longati *et al*.^31^, was used in the context of a sugarcane 1G-2G ethanol biorefinery. This methodology, called retro-techno-economic analysis (RTEA), turns the normal techno-economic analysis (TEA) approach upside down. In the RTEA, instead of analyzing a specific operational condition for a predefined process and evaluating its economic feasibility, a minimum economic performance is defined (such as setting the net present value, NPV, equal zero). Then, windows of economically feasible operational conditions are generated, which can be used to obtain performance target values for the main process variables^30,31^.

This paper describes the techno-economic impact of the use of soybean protein as an additive in the enzymatic hydrolysis of sugarcane bagasse, in the context of a biorefinery producing 1G-2G ethanol. An initial set of experiments was carried out to define process variables including solids loading, soybean protein concentration, enzyme dosage, and hydrolysis time, using statistical experimental design methodology as a tool. The techno-economic analysis employing the RTEA methodology allowed the definition of some performance targets to be achieved experimentally, in order to make the use of soybean protein economically feasible.

## Results and Discussion

### Effects of solids loading and soybean protein concentration

Selection of the process parameters of the enzymatic hydrolysis reaction, such as sugarcane bagasse loading and additive concentration, is very important in order to obtain high cellulose conversion, glucose release, and process gain (% increase in glucose release provided by soybean protein). These response variables are crucial for evaluating the performance of the additive during the saccharification, avoiding excessive use of it in the process. Table 1 presents the central composite rotatable design (CCRD) matrix, with the values of the independent variables (coded and uncoded) and the response variables (glucose release, cellulose conversion, and process gain). For this set of experiments, the enzyme dosage was fixed at 5 FPU/g solids, in order to avoid a high loading of enzymes masking the effect of soybean protein during the hydrolysis reaction.

**Table 1:** Central composite rotatable design (CCRD) matrix with coded and uncoded (in parentheses) values of the independent variables solids loading (% w/w) and soybean protein loading (% w/w). The response variables analyzed were glucose release (g/L), cellulose conversion (%), and process gain (%). The hydrolysis was carried out with an enzyme dosage of 5 FPU/g dry biomass and 24 h of reaction.

Run	Solids loading (% w/w)		Soybean protein loading (% w/w)		Glucose (g/L)	Conversion (%)	Gain (%)
1	−1	(10)	−1	(4)	19.88	31.94	14.03
2	+1	(20)	−1	(4)	29.08	23.37	2.63
3	−1	(10)	+1	(12)	22.92	36.83	31.49
4	+1	(20)	+1	(12)	30.39	24.42	7.24
5	−1.41	(8)	0	(8)	16.28	32.72	35.08
6	+1.41	(22)	0	(8)	25.30	18.48	−7.07
7	0	(15)	−1.41	(2.4)	24.99	26.78	7.98
8	0	(15)	+1.41	(13.6)	28.88	30.88	24.50
9	0	(15)	0	(8)	27.40	29.36	18.38
10	0	(15)	0	(8)	28.02	30.02	21.05
11	0	(15)	0	(8)	28.30	30.32	22.27

The influence of the independent variables solids loading (SL) and soybean protein loading (SP) on the response variables was evaluated by examination of the significance (p < 0.05) of their individual effects and their interaction, obtained by analysis of variance (ANOVA) for quadratic models (Table 2). For all the responses, the linear term of SL had the greatest effect, with a positive influence on glucose release and negative influences on cellulose conversion and process gain (these variables decreased as the solids loading increased). The linear term of SP had significant positive effects for all the dependent variables. The terms that were not statistically significant were not considered further.

**Table 2:** Coefficient values and statistical analysis for glucose concentration, cellulose conversion, and process gain. R^2^ is the coefficient of determination, SL is the solids loading, and SP is the soybean protein loading. Significant parameters (p ≤ 0.05) are indicated (*).

	*Glucose*		*Conversion*		*Gain*	
	Coefficient	p-value	Coefficient	p-value	Coefficient	p-value
Mean	27.907*	0.000	29.900*	0.000	20.570*	0.003
SL	7.357*	0.002	−10.280*	0.001	− 23.815*	0.003
SL^2^	−6.257*	0.004	−3.338*	0.015	−7.199	0.051
SP	2.442*	0.017	2.935*	0.014	11.358*	0.014
SP^2^	−0.142	0.750	−0.108	0.819	−4.964	0.097
SL × SP	−0.865	0.201	−1.920	0.060	−6.425	0.084
R^2^	0.952		0.955		0.870	
F-value	45.742		49.004		26.507	
F_cal_/F_listed_	10.515		11.265		5.943	

**Figure 1:**
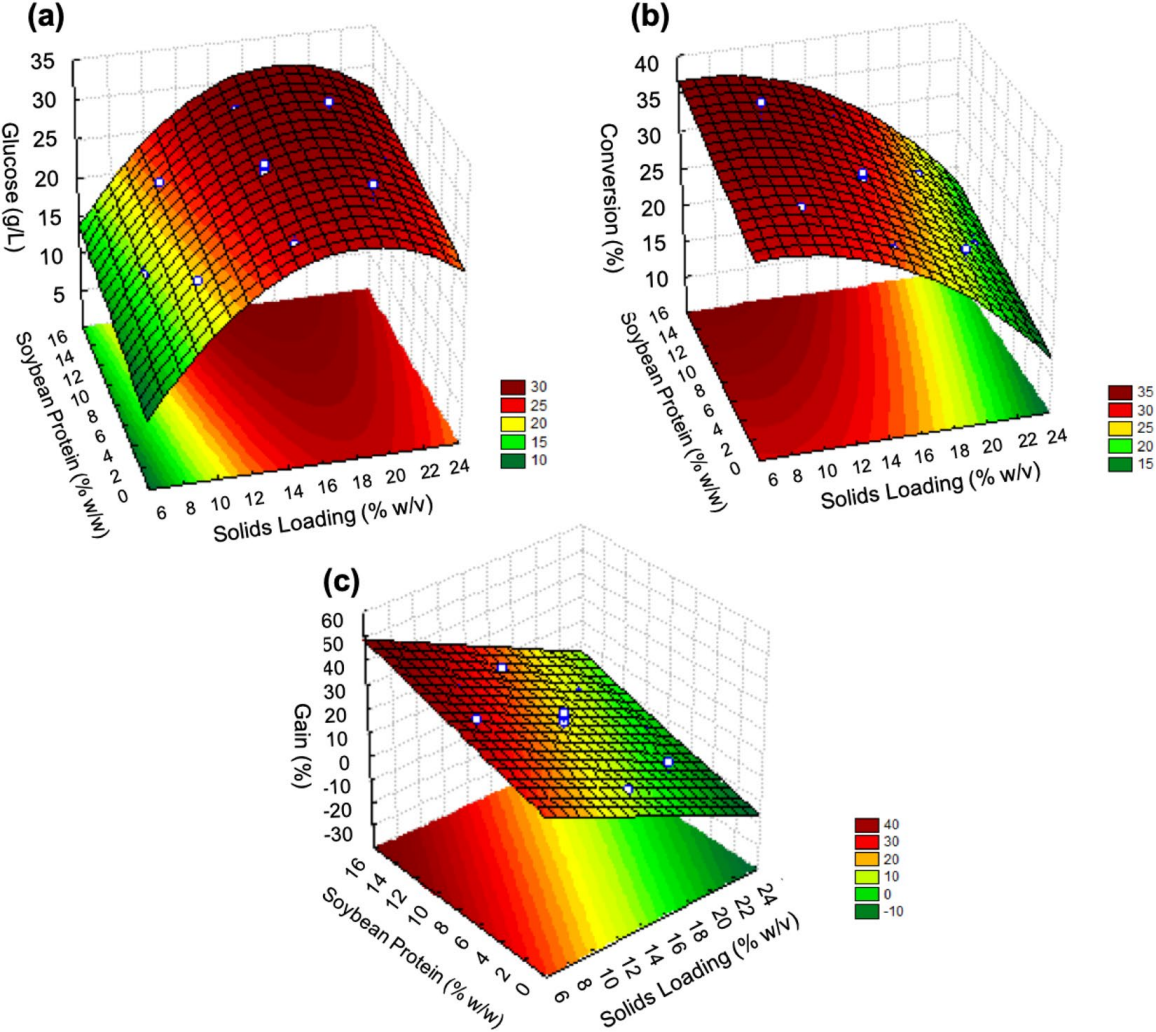
Response surface for each dependent variable analyzed: (**a**) glucose release (g/L), (**b**) cellulose conversion (%), and (**c**) process gain (%). The experiments were carried out at 50 °C, with a fixed enzyme loading of 5 FPU/g dry bagasse and a hydrolysis time of 24 h.

After definition of the significant terms, the models were evaluated using the correlation coefficient (R^2^) and the Fischer test (F-test), together with the generation of response surfaces (Fig. 1). All the models presented satisfactory R^2^ and for each variable, the calculated values of F were higher than the listed values (Table 2). This implied that the surface response equations obtained for each variable (glucose release, cellulose conversion, and process gain, represented by Eqs. 1, 2, and 3, respectively), for coded values of SL and SP, were statistically significant. Therefore, they provided satisfactory representations of the way that the independent variables (solids loading and additive concentration) affected the responses.

1 $$Glucose\left(\frac{g}{L}\right)=27.84+7.357\times SL-6.215\times S{L}^{2}+2.442\times SP$$

2 $$Conversion( \% )=29.849-10.279\times SL-3.306\times S{L}^{2}+2.935\times SP$$

3 $$Gain( \% )=16.144-23.815\times SL+11.358\times SP$$

The response surface showed that increases of the solids and soybean protein loadings led to increased glucose release (Fig. 1a). For cellulose conversion (Fig. 1b) and process gain (Fig. 1c), high concentrations of soybean protein had the same positive effect, enhancing these response variables. However, increase of the solids loading led to decreases of cellulose conversion and process gain. These results were consistent with previous findings that increase of the solids loading enhanced glucose release, while it negatively affected cellulose conversion^14,32,33^. This decrease in conversion could be attributed to problems in mixing and mass transfer, together with the inhibition of cellulases by the products formed during the hydrolysis, which affected saccharification performance when using high solids loadings^32,33^.

**Figure 2:**
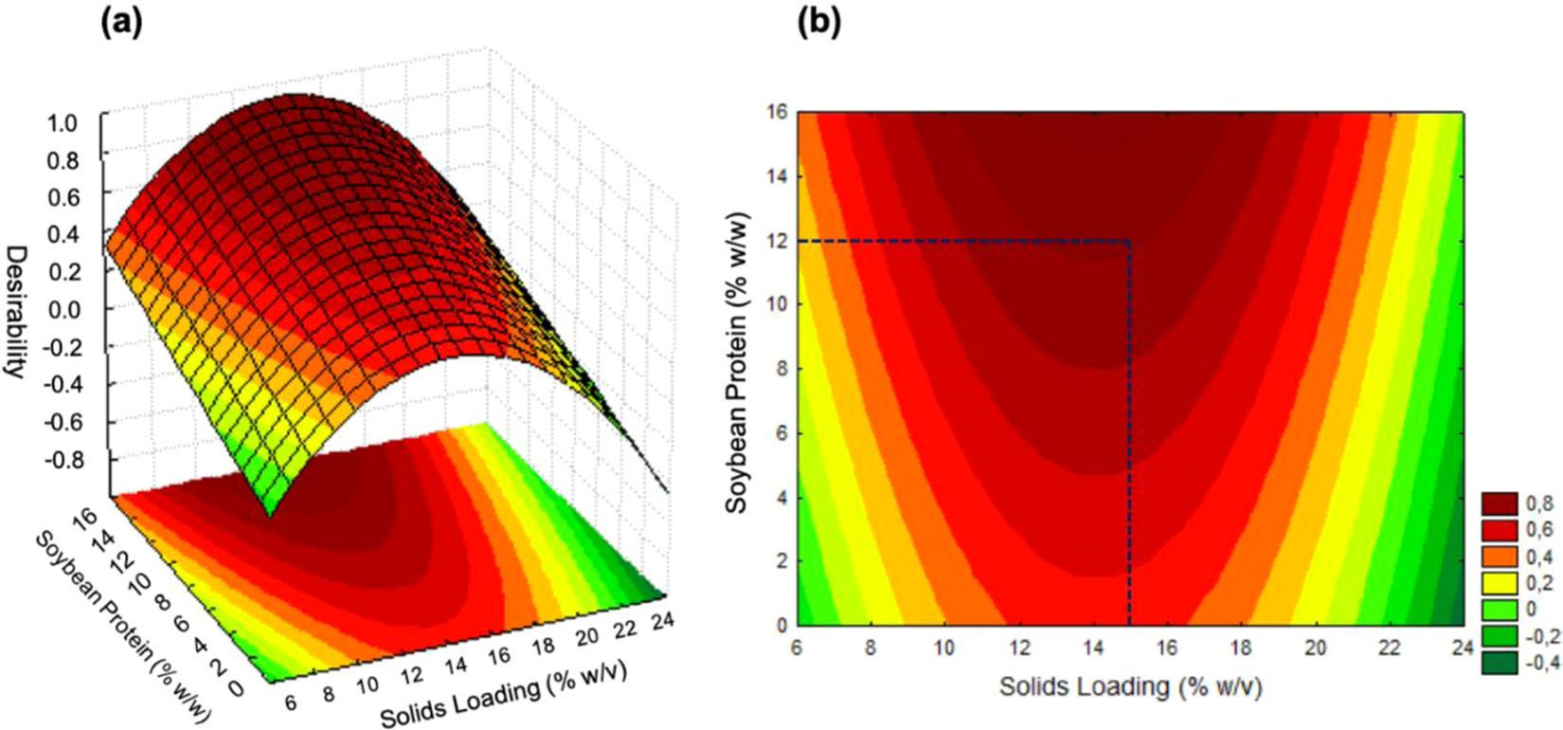
Response surface (**a**) and contour plot (**b**) for the desirability function, showing the values of the solids and soybean protein loadings that simultaneously ensured high glucose release, cellulose conversion, and process gain.

After definition of the statistical models by means of experimental design, the desirability function was employed in order to determine the solids and soybean protein loading values at which high values for glucose release, cellulose conversion, and process gain were obtained simultaneously. This analysis was performed because all these variables were important for ensuring the economic feasibility of 2G ethanol production using soybean protein as an additive. Figure 2 presents the response surface and the desirability contour plot, where values closer to 1 represent more desirable regions (high values for glucose release, cellulose conversion, and process gain).

It is important to emphasize that a high solids loading (≥15%) is a desirable process condition for the hydrolysis reaction, since it leads to higher glucose release and consequently higher 2G ethanol productivity^32–34^. The analysis of Fig. 2 showed that the solids loading and soybean protein concentration that enabled operation in a desirable region with a high solids loading were 15% (w/w) of sugarcane bagasse and 12% (w/w) of additive, as indicated by the dark blue dashed lines in the contour plot (Fig. 2(b)). The additive concentration was set at 12% (w/w), because this was the lowest possible value within the desirable region, avoiding excessive use of the additive during the hydrolysis.

After defining these parameters, validation of the statistical models represented by Eqs. 1, 2, and 3 was performed, with 15% solids (SL) and 12% additive (SP) having coded values of 0 (zero) and +1, respectively. Table 3 presents the values predicted by the models for the three response variables, together with the experimental data obtained by performing the enzymatic hydrolysis under these conditions. Taking into account the standard deviations associated with each experimental response variable, it can be seen that the experimental values were very close to the values predicted by the models. Therefore, these values of sugarcane bagasse loading and soybean protein concentration, defined by application of the desirability function, were selected in the next experimental steps.

**Table 3:** Experimental design model validation. The predicted values were obtained using the model equations for each response variable. The experimental values are presented as average ± standard deviation for triplicates.

Response variable	Predicted value	Experimental value
Glucose (g/L)	30.28	30.5 ± 0.4
Conversion (%)	32.78	32.7 ± 0.4
Gain (%)	27.50	26.9 ± 1.4

### Effect of reaction time and enzyme dosage

**Figure 3:**
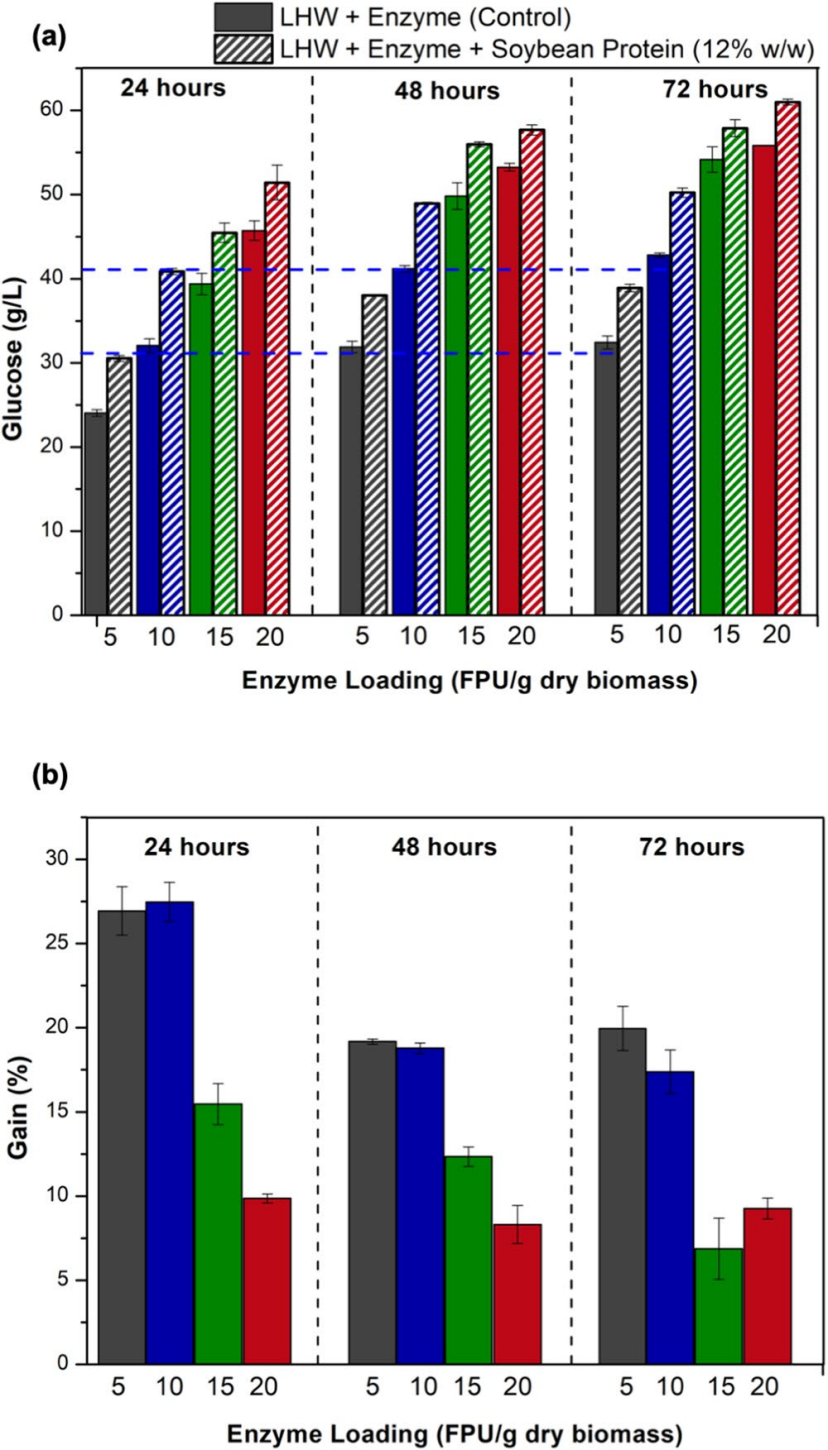
Effect of soybean protein (12% w/w) over time (24, 48, and 72 h), using different enzyme loadings (5, 10, 15, and 20 FPU/g dry bagasse) and a fixed sugarcane bagasse loading of 15% (w/w). (**a**) Glucose release (g/L), where the bars with solid colors show the control (hydrolysis without additive), while the hatched bars show the glucose released after the addition of soybean protein. (**b**) Process gain (%) provided by the addition of soybean protein.

Based on the statistical experimental design, the solids loading and the soybean protein concentration were fixed at 15% (w/w) and 12% (w/w), respectively. The effect of the additive was then assessed for 24, 48, and 72 h of hydrolysis, using 5, 10, 15, and 20 FPU/g dry bagasse of the commercial Cellic CTec3 enzyme cocktail. Figure 3(a) shows the glucose released in the absence (control run) and presence of the additive, while Fig. 3(b) shows the process gain obtained by the addition of soybean protein. It can be seen that the addition of soybean protein increased the release of glucose, for all the conditions evaluated.

The blue dashed lines in Fig. 3(a) highlight an interesting result, where the hydrolysis for 24 h using the enzyme cocktail at 5 FPU/g and 12% (w/w) of additive released essentially the same amount of glucose (30.6 g/L) found for the control after 48 and 72 h of saccharification, also using the enzyme cocktail at 5 FPU/g (31.8 and 32.4 g/L, respectively). The same trend was observed in the assays using the enzyme cocktail at 10 FPU/g. Hence, the addition of soybean protein allowed the hydrolysis time to be reduced by up to 66%, while maintaining the same process efficiency, which would be beneficial in terms of the economics of the process. Furthermore, 24 h of hydrolysis using the enzyme cocktail at 5 FPU/g, in the presence of the additive, resulted in a glucose concentration of 30.6 g/L, while 32 g/L of glucose was obtained for the control with 10 FPU/g. Therefore, use of the soybean protein allowed the enzyme load to be reduced by 50%, while achieving the same yield of sugar. The same trends were observed for comparison of 10 FPU/g, with soybean protein, and the control at 15 FPU/g, as well as for 15 FPU/g, with additive, and the control at 20 FPU/g. These results implied enzymatic loading reductions of 33 and 25%, respectively. Reduction of the amount of enzyme used during the saccharification process is extremely important for its economic feasibility, due to the high costs of enzymes, which have important impacts on the minimum selling price of 2 G ethanol^4,6^. It should also be pointed out that soybean protein has a much lower cost than cellulolytic enzymes^5^.

Figure 3(b) shows the percentage increase in glucose release (process gain) provided by the addition of soybean protein, in comparison to the control. For all the times and enzyme dosages evaluated, the presence of the additive increased the sugar yield, with the greatest enhancement for a saccharification time of 24 h and enzyme loadings of 5 and 10 FPU/g bagasse, where the gains were higher than 26%. When low enzyme dosages are used and no additive is employed in the process, the occurrence of nonproductive adsorption significantly decreases the amount of free cellulase available to hydrolyze the biomass, which substantially reduces the conversion. Hence, the most positive effects (gains) are observed when additives are used with low enzyme loadings^35–37^.

The effects of soybean protein during the hydrolysis of sugarcane bagasse for different times, using the Cellic CTec2 enzymatic cocktail, were reported by Brondi *et al*.^11^ and Florencio *et al*.^21^. Both previous studies found that the highest gains, compared to the control, were achieved for 24 h assays. Florencio *et al*.^21^ showed that the hydrolysis of liquid hot water pretreated sugarcane bagasse (15% w/w) during 24 h presented the highest gains when enzyme loadings of 5 and 10 FPU/g dry biomass were used, together with an additive loading of 12% (w/w). This was in agreement with the data presented here using Cellic CTec3, which is a more recent commercial cocktail and contains a different set of enzymes in its composition^38^.

The hydrolysis of liquid hot water pretreated sugarcane bagasse using one of the commonest additives, namely the surfactant Tween 80, was described by Yu *et al*.^39^. Saccharification of 5% (w/v) bagasse was performed using a commercial cellulase at a loading of 15 FPU/g dry solids. For 24 h of hydrolysis, no significant effect was observed after the addition of 0.5 v/v of Tween 80. However, after 72 h, an increase of 34% was observed, compared to the control. Comparison of these results with the present findings suggests that soybean protein acts faster than Tween 80 in improving the hydrolysis.

### Effect of soybean protein using a bench-scale reactor

After definition of the operational parameters for saccharification (15% w/w sugarcane bagasse loading, 12% w/w soybean protein concentration, 10 FPU/g dry substrate, and 24 h of reaction), assays were performed using a bench-scale reactor (a 0.5-L stirred tank equipped with two Elephant Ear impellers) as a system closer to industrial reality^40^. An enzyme dosage of 10 FPU/g was chosen, since it provided a higher cellulose conversion than a dosage of 5 FPU/g.

**Figure 4:**
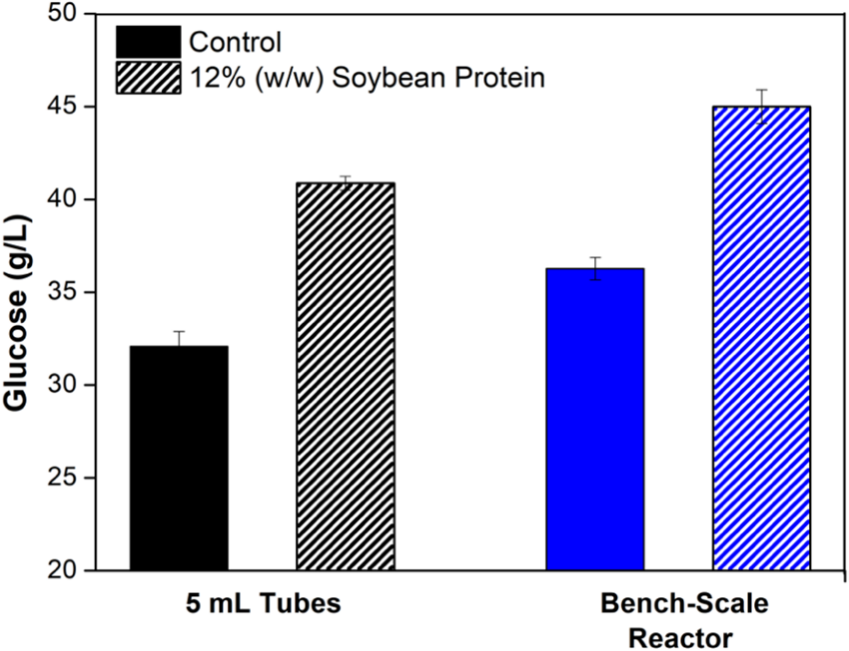
Glucose released during the enzymatic hydrolysis in the bench-scale reactor compared to the 5 mL tube. The hydrolysis was carried out for 24 h and 50 °C, using a sugarcane bagasse loading of 15% (w/w), an enzyme dosage of 10 FPU/g dry bagasse, and 12% (w/w) of soybean protein. The control (solid colors) represents the hydrolysis without additive.

The addition of soybean protein increased the glucose released (Fig. 4), with the gain observed for the bench-scale reactor being similar to that found using 5 mL flasks (25 and 26%, respectively). It is important to highlight that reactor conditions such as agitation velocity and the feed mode could be optimized further, improving the release of glucose, cellulose conversion, and even the positive gain provided by the addition of soybean protein. For example, Santos-Rocha *et al*.^41^ reported a 19.7% increase in enzymatic conversion of hydrothermally pretreated sugarcane straw cellulose, when the operational mode was changed from batch to fed-batch (59.65 and 71.43% cellulose conversion, respectively), while maintaining fixed the hydrolysis time (72 h), enzyme dosage (10 FPU/g substrate), and final solids loading (30% w/v).

### Techno-economic analysis

After confirming the positive effect of soybean protein under experimental conditions closer to those found in industry, a techno-economic analysis was performed, in order to evaluate the feasibility of using soybean protein addition in the context of a biorefinery producing 1G-2G ethanol from sugarcane^31^. Initially, a local sensitivity analysis was performed for the conditions defined experimentally in order to establish which variable (loadings of enzyme, bagasse or additive) most affected the process. This sensitivity analysis showed that the enzyme dosage was the variable with the greatest negative effect on the hydrolysis (−3.61), in agreement with the findings of Longati *et al*.^31^, followed by the soybean protein concentration (−1.00) and then the bagasse loading (−0.74), which also negatively affected the process.

**Figure 5:**
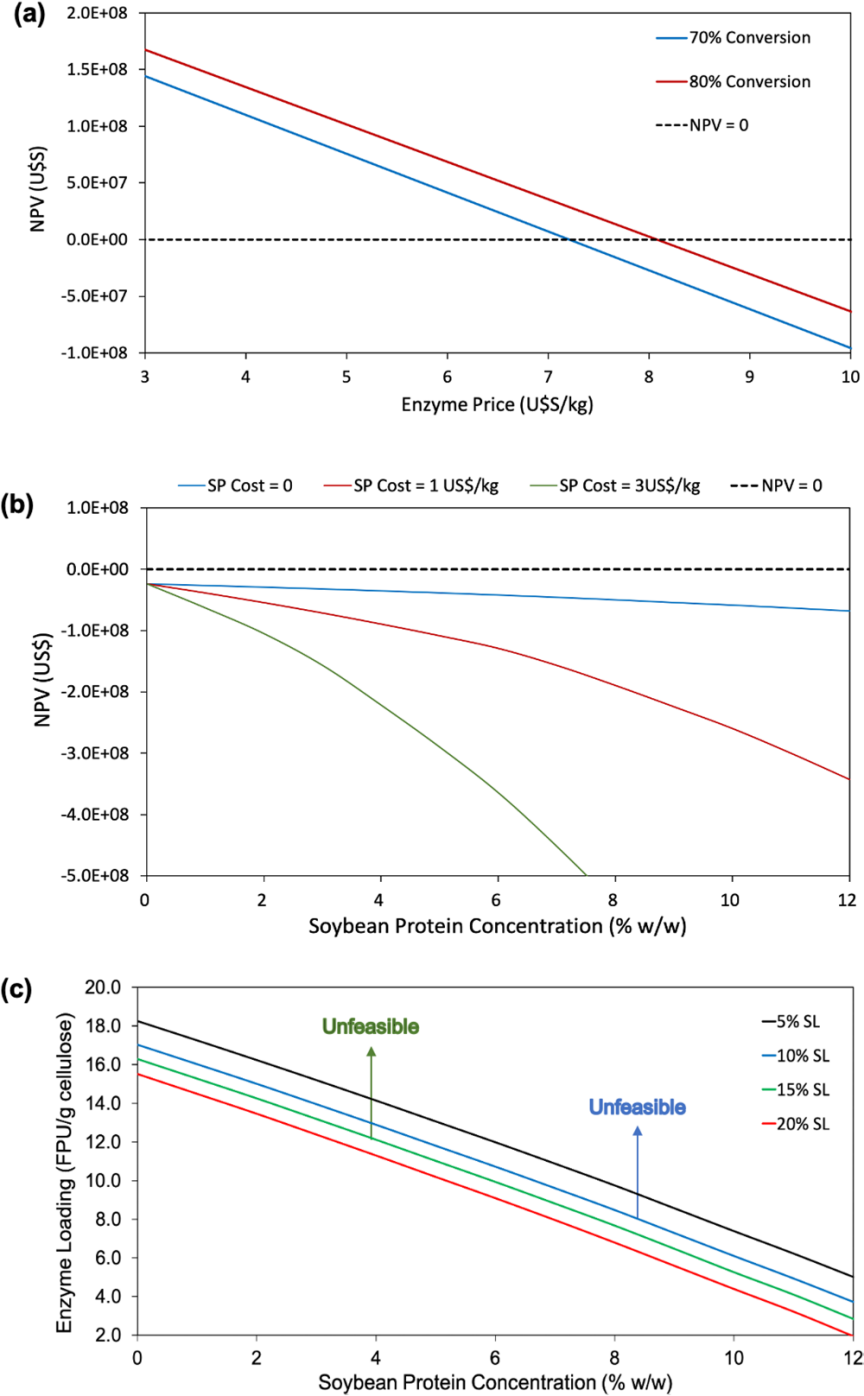
Techno-economic analysis for a hydrolysis time of 24 h. (**a**) Effect of enzyme price on the process NPV, considering a fixed enzyme loading of 18 FPU/g cellulose, 12% (w/w) of soybean protein, and 15% (w/w) of solids in the hydrolysis reactor. Cellulose conversions of 70 and 80% were evaluated. (**b**) Evaluation of the effect of soybean protein cost (0, 1, and 3 US$/kg) on the NPV of the biorefinery. For this analysis, solids loading, hydrolysis time, enzyme dosage, and cellulose conversion were fixed at 15% (w/w), 24 h, 18 FPU/g cellulose, and 80%, respectively. The black dashed line represents NPV = 0. (**c**) Feasibility curves obtained by the RTEA evaluating the effects of the variables solids loading in the hydrolysis reactor and soybean protein concentration on the maximum enzyme dosage required for the biorefinery to have a null net present value (NPV = 0). This analysis was carried out assuming that the soybean protein costed 1.00 US$/kg for the biorefinery. Conversion was set at 80% and solids loading (SL) of 5, 10, 15 and 20% (w/w) were analyzed. The regions of feasibility are below the curves.

The substantial negative impact of the enzymatic cocktail on the process economics was due to its high cost. Therefore, lower amounts of enzymes were required for feasibility^4,5^. For the biorefinery simulation, the enzymatic cocktail price was assumed to be 10.14 US$/kg^5^. The effect of the enzyme price on the process NPV was evaluated (Fig. 5(a)), considering a hydrolysis condition closer to that defined experimentally, employing 24 h of reaction, 15% (w/w) of solids in the saccharification reactor, 12% (w/w) of additive, and a fixed enzyme loading of 18 FPU/g cellulose (~10 FPU/g dry bagasse). Biomass conversions of 70 and 80% were considered since the experimental conversion value of 48% resulted in negative NPV for all evaluated enzyme prices. Besides, biomass conversions of 70 and 80% had been previously reported for the hydrolysis of sugarcane bagasse using an optimized bioreactor^42^.

As shown in Fig. 5(a), an enzyme price of 10.14 US$/kg resulted in negative NPV for both 70 and 80% conversions. However, when the prices became lower than 8 and 7.2 US$/kg for the conversions of 80 and 70%, respectively, positive NPV was obtained. These results showed that decreasing the cost of the enzymes could greatly contribute to process feasibility. In addition, higher conversions than obtained experimentally (48%) were necessary, which could be achieved by increasing the saccharification efficiency of the enzymatic cocktails.

In the next step, an evaluation was made of the effects of soybean protein additive cost (0, 1, and 3 US$/kg) and concentration on the NPV (Fig. 5(b)). Cellulose conversion was fixed at 80%, the enzyme dosage was 18 FPU/g cellulose (~10 FPU/g dry bagasse), the solids loading was 15% (w/w) and the enzymatic cocktail price was kept at 10.14 US$/kg^5^. Increasing both the additive cost and its concentration negatively impacted the biorefinery, resulting in negative NPV for all cases. This result is in agreement with the data shown in Fig. 5(a), where for 80% of conversion, the feasibility is reached only with an enzyme cost lower than 8 US$/kg.

It is important to note that according to the analysis performed here, an average market price of 3 US$/kg for isolated soybean protein, as reported by Brondi *et al*.^11^, would be too high to make adding soybean protein during the enzymatic saccharification economically feasible. However, synergy between a soybean oil/biodiesel refinery and a 1G-2G ethanol plant could be exploited, where the saccharification additive would not be a commercial soybean protein, but instead would be a lower value byproduct of soybean oil extraction. Consequently, the use of market prices for soybean protein results in overestimation of the cost of the additive.

### Retro-techno-economic analysis

In order to determine the windows of economically feasible operational conditions to obtain the performance target values for the main process variables, the retro-techno-economic analysis (RTEA) methodology was employed^30,31^. For that, the net present value (NPV) of the industrial plant was set at zero and the effects of solid mass fraction in the hydrolysis reactor, soybean protein concentration and enzyme loading were analyzed. Firstly, the interaction between the performance indices and the operational variables was calculated, considering the effects on the economics of the process. The cost of the soybean protein was assumed to be US$1.0/kg of protein, given the possibility of integration between soybean biodiesel and sugarcane bioethanol production processes^43^, where the soybean protein would be a byproduct of the biorefinery.

Figure 5(c) presents the feasibility curves for different solids loading (SL) of 5, 10, 15 and 20% (w/w), with a fixed biomass conversion of 80%, where it was possible to evaluate the effect of the enzyme loading over different soybean protein concentrations (from zero to 12%). These contour curves, called isoeconomic curves, represent the minimum necessary conversions, given certain conditions of solids loading, enzyme amount, and additive loading, for an economically feasible operation (NPV = 0). The feasible regions lie below the curves (with NPV > 0). For this evaluation, the hydrolysis time was fixed at 24 h. An example of how this figure could be interpreted is that for a cellulose conversion of 80%, a solid loading of 15% (w/w) and an additive loading of 6% (w/w), a maximum enzyme dosage of 10 FPU/g cellulose (~5.6 FPU/g dry bagasse) is required for the process to become economically feasible. For this set of conditions, enzyme loadings higher than 10 FPU/g cellulose implied unfeasibility.

As can be seen (Fig. 5(c)), for a fixed bagasse loading and cellulose conversion value, an increase in soybean protein concentration in the process requires the enzyme loading to be reduced, in order to maintain the feasibility of the process. When soybean protein was added to the process, it increased the costs of the biorefinery, consequently requiring a reduction of the enzyme loading, in order to maintain NPV = 0. This could be explained by a traditional techno-economic analysis of the data shown in Table 4, which presents an evaluation of the effect of soybean protein addition on some of the main parameters of biorefinery economics, considering fixed values for the sugarcane bagasse loading, enzyme dosage, cellulose conversion, and hydrolysis time.

**Table 4:** Economic analysis of soybean protein addition, in the context of a biorefinery. For this evaluation, the sugarcane bagasse loading in the hydrolysis reactor was fixed at 15% (w/w), the hydrolysis time was 24 h, the enzyme dosage was 18 FPU/g cellulose, and the cellulose conversion was fixed at 80%. The cost of the additive was considered to be US$1.0/kg.

*SP (% w/w)*	0	5	12
Fraction of bagasse for 2G ethanol	0.65	0.73	0.89
CAPEX - Pretreatment (US$)	7.25E + 07	7.73E + 07	8.67E + 07
CAPEX - Hydrolysis (US$)	2.01E + 07	2.21E + 07	2.63E + 07
CAPEX - Cogeneration (US$)	8.53E + 07	8.84E + 07	9.52E + 07
OPEX - Pretreatment (US$)	4.70E + 05	5.27E + 05	6.43E + 05
OPEX - Hydrolysis (US$)	5.42E + 07	7.62E + 07	1.23E + 08
OPEX - Cogeneration (US$)	2.22E + 07	2.36E + 07	2.66E + 07
Pretreatment – water flow (kg/h)	6.41E + 05	7.18E + 05	8.77E + 05
Pretreatment – ammonia flow (kg/h)	9.02E + 02	1.01E + 03	1.23E + 03
Hydrolysis – water flow (kg/h)	2.41E + 05	2.66E + 05	3.19E + 05
Hydrolysis – enzyme flow (kg/h)	8.66E + 03	9.70E + 03	1.18E + 04
Hydrolysis – soybean protein (kg/h)	0	3.28E + 03	1.04E + 04
Anhydrous ethanol production (m^3^/h)	99.52	102.09	107.40
W net (kW)	7.48E + 04	8.08E + 04	9.40E + 04
Revenue (US$)	2.73E + 08	2.81E + 08	2.99E + 08
NPV (US$)	−2.37E + 07	−1.08E + 08	−3.42E+08

When soybean protein was added to the process, the sugarcane bagasse fraction sent to the production of 2 G ethanol increased from 0.65 to 0.73 and 0.89, using 0, 5, and 12% of additive, respectively (Table 4). This increase of the bagasse fraction was because after the hydrolysis, the unhydrolyzed material, together with the additive, was sent to the cogeneration sector, where it was combusted to generate bioelectricity and steam for the biorefinery. Due to the presence of protein, a smaller amount of sugarcane bagasse (a byproduct of 1G ethanol production) needs to be burned in the boilers, in order to supply the energy demand of the industrial plant. Therefore, the amount of bagasse available for the 2G ethanol production process is increased. The addition of soybean protein and the greater amount of biomass available for the 2G sector increased anhydrous ethanol production by 8%, from 99.52 m^3^ h^−1^, when no additive was used, to 107.40 m^3^ h^−1^, when 12% (w/w) protein was added. This addition also increased the bioelectricity surplus that could be sold by the biorefinery.

Although the additive had positive impacts in terms of improving ethanol and electricity production, the increase of the soybean protein concentration had a clear negative impact on the biorefinery NPV, because it increased the capital expenditure (CAPEX) and operational expenditure (OPEX). The availability of more bagasse for the 2G sector would require larger reactors for the pretreatment and hydrolysis processes, hence increasing the CAPEX of these sectors. Furthermore, in order to maintain the cellulose conversion and solid loading fixed, the pretreatment and hydrolysis of a greater quantity of biomass would require more water, ammonia (used after the pretreatment, in order to regulate the hydrolysis pH), and enzymes, hence increasing the biorefinery OPEX. There would also be increased steam and energy demands, increasing the costs of the cogeneration sector. For the case using 12% of soybean protein in the hydrolysis, the increases of ethanol production and bioelectricity surplus provided by the additive would not be sufficient to ensure an economically feasible process, due to the increases of the biorefinery CAPEX and OPEX.

For a fixed concentration of additive, an increase in the solids loading decreased the maximum enzyme load that would make the process economically feasible (Fig. 5(c)). Common sense suggests that high solid loadings (once problems such as those related to mixing and mass transfer resistance are solved) would be advantageous for the hydrolysis, since the amount of glucose released is increased. Nevertheless, the results showed that when the overall biorefinery was considered, an increase in the bagasse loading in the saccharification reactor had a negative effect (for fixed amounts of enzyme and additive, increasing the solid fraction in the hydrolysis reactor requires higher conversions to maintain the feasibility of the biorefinery). This was because an increase of the sugarcane bagasse loading implied an increase of the enzyme dosage required to maintain a fixed conversion in the hydrolysis step. However, due to the cost of the enzymatic cocktails, this would negatively affect the process NPV.

### Performance targets

The retro-techno-economic analysis enabled the establishment of performance targets to be achieved experimentally in order to make the integrated biorefinery economically feasible. Performance targets aiming to increase the biomass conversion, decrease soybean protein concentration and/or its price, and reduce the enzyme loading and/or its price should be pursued. An example of how these targets can be obtained by the analysis of Fig. 5(c) is that the hydrolysis with 12% (w/w) of additive, 15% (w/w) of bagasse loading and 18 FPU/g of cellulose (~10 FPU/g dry bagasse) of enzyme, with 80% of conversion, results in an unfeasible process. However, if the R&D team were able to create an enzymatic cocktails more efficient, that would be able to perform the saccharification with 10 FPU/g cellulose (~5.6 FPU/g of dry bagasse) in the presence of soybean protein (6% w/w), maintaining the same yield, the biorefinery would became economically feasible at these conditions. Therefore, if these performance targets were achieved, the result would be a significant contribution to making the production of lignocellulosic ethanol economically feasible.

## Conclusions

A systematic study employing techno-economic analysis was performed to evaluate the effects of operational conditions during sugarcane bagasse enzymatic hydrolysis reactions with soybean protein as an additive. The experimental results demonstrated that this additive could effectively enhance saccharification of the biomass. Loadings of solids and soybean protein, enzyme dosage, and hydrolysis time were defined experimentally, so that the glucose released during saccharification of liquid hot water pretreated sugarcane bagasse increased by up to 26%. The evaluation of these conditions using RTEA allowed the definition of some important performance targets to be achieved experimentally, in order to make the integrated biorefinery economically feasible. These performance targets were reductions of the cost and concentration of the additive used in the hydrolysis, increased conversion in the reactor, and decreased enzyme loading. For instance, the RTEA showed that increase the biomass conversion to 80% and reduce the enzyme loading to 5.6 FPU/g would make the biorefinery economically feasible in the presence of soybean protein. If these performance targets were achieved, the use of soybean protein would also lead to increases of the yield of bioethanol and the export of bioelectricity. The results here obtained indicated that if soybean protein was used as an additive during the enzymatic saccharification of lignocellulosic biomass, and if the performance targets were achieved, then the additive would help to enhance the saccharification yield, hence overcoming one of the main technological bottlenecks of the 2G ethanol production process.

## Methods

### Materials

Sugarcane bagasse (kindly donated by Ipiranga sugarcane mill, São Paulo State, Brazil) was used as lignocellulosic biomass. The bagasse was submitted to a liquid hot water (LHW) pretreatment for 10 min, at 195 °C, in a 5-L reactor (Model 4580, Parr Instruments), using a solids loading of 10% (w/v). After the pretreatment, the substrate (which was not washed) was dried at room temperature until the moisture content decreased to below 10%. The biomass was then milled to a particle size (d_p_) ≤1 mm. Chemical characterization was performed as described by Gouveia *et al*.^44^. The composition of the LHW pretreated sugarcane bagasse (% w/w) was 56% glucan, 6% pentosan, 29% lignin, and 4% ash. Soybean protein isolate (protein content ≥90%, Bremil, Rio Grande do Sul, Brazil) was used as the additive. The commercial enzymatic cocktail Cellic CTec3 (Novozymes, Paraná, Brazil) was used in the enzymatic hydrolysis experiments. The cellulolytic activity (FPU/mL) was determined according to the methodology proposed by Ghose^45^.

### Enzymatic hydrolysis

The enzymatic hydrolysis experiments were carried out at 50 °C, using citrate buffer (50 mM, pH 4.8), in 5 mL tubes, under different conditions of solids loading, enzyme dosage, soybean protein concentration, and hydrolysis reaction time. The experiments were carried out in an incubator, with rotary mixing at 30 rpm, in the presence and absence (control runs) of soybean protein. The glucose released was determined using a glucose enzymatic assay kit (Labtest, Brazil). All the experiments were performed in triplicate.

Cellulose conversion (%) and process gain (%) in the batch runs were calculated according to Eqs. (4) and (5), respectively. For the conversion, m_g_ is the glucose mass released after the hydrolysis, 0.9 is the conversion factor, m_b_ is the bagasse mass, and t_c_ is the cellulose content. The process gain was determined as the ratio between the amounts of glucose released in the presence of the additive and without additive (control sample).

4 $$Conversion( \% )=\frac{{m}_{g}\times 0.9}{{m}_{b}\times {t}_{c}}\times 100 \% $$

5 $$Gain( \% )=\left(\frac{Glucose\,concentration(with\,additive)}{\,Glucose\,concentration(without\,additive)}-1\right)\times 100 \% $$

### Experimental design methodology

A central composite rotatable design (CCRD) with 11 runs was performed in order to evaluate the effects of the independent variables solids loading (% w/w) and soybean protein loading (% w/w), during enzymatic hydrolysis of the LHW pretreated sugarcane bagasse, considering the response variables glucose release (g/L), cellulose conversion (%), and process gain (%). Table 1 shows the factors and levels analyzed, together with the responses. The data were submitted to analysis of variance (ANOVA), with a significance level of 95% (p = 0.05). The effects of the independent variables on the response variables were evaluated by response surface analysis. The surface plots and the ANOVA analysis were performed using STATISTICA v. 13.3 software (StatSoft - https://www.tibco.com/resources/product-download/tibco-statistica-trial-download-windows). For the experimental design, the hydrolysis time was fixed at 24 h and the enzyme dosage was 5 FPU/g dry substrate.

The desirability function^46^ was used to identify the solids and soybean protein loadings that would simultaneously provide high values for glucose release, cellulose conversion, and process gain. This analysis consisted of converting each of the three response variables into a single desirability function (d_i_), in the range from 0 to 1 (0 ≤ d_i_ ≤ 1). When d_i_ is close to 0, it represents an undesirable value, while d_i_ close to 1 represents a more desirable response. The global desirability function (D) is calculated as the geometric mean of the three individual desirabilities: D = (d_1_ × d_2_ × d_3_)^1/3^. This analysis was also performed with the STATISTICA software. After definition of the most desirable values for the solids and soybean protein loadings, the statistical models previously obtained for each response variable were validated for this condition (15% (w/w) of bagasse, 12% (w/w) of additive, 5 FPU/g substrate, and 24 h of hydrolysis). The values for glucose release, cellulose conversion, and process gain obtained experimentally were then compared to the values predicted by the statistical model. This set of experiments was performed in triplicate in 5 mL tubes, at 50 °C, in an incubator with rotary mixing at 30 rpm. The data were presented as average ± standard deviation.

### Time profile evaluation

After defining the loadings for solids (15% w/w) and soybean protein (12% w/w), using the statistical experimental design as a tool, these variables were fixed in order to perform the time profile assays. In this step of the study, different hydrolysis times (24, 48, and 72 h) and enzyme loadings (5, 10, 15, and 20 FPU/g dry bagasse) were evaluated in order to find the condition for these two variables that resulted in the most positive effect of the soybean protein on the response variables glucose release and process gain. These experiments were performed in triplicate in 5 mL tubes, at 50 °C, in an incubator with rotary mixing at 30 rpm. The data were presented as the average ± standard deviation.

### Bench-scale reactor

After definition of the solids and additive loadings, enzyme dosage, and hydrolysis time, using 5 mL tubes, these conditions were tested using a bench-scale reactor (with and without additive). The experiments were performed using a 0.5-L working volume stirred tank reactor with an internal diameter of 0.085 m and a total height of 0.140 m. The reactor was equipped with two three-blade Elephant Ear (EE) impellers with diameters of 0.040 m^47^. The pH and temperature were the same as used with the 5 mL flasks and the stirring speed was 250 rpm. The experiments were performed in triplicate and the data were presented as the average ± standard deviation.

### Retro-techno-economic analysis

The techno-economic analysis was performed in the context of a biorefinery processing sugarcane bagasse and producing 1 G and 2 G ethanol, besides bioelectricity. The models for this biorefinery were presented by Longati *et al*.^31^ and the same configuration was used here to evaluate the effect of soybean protein addition. The software used was EMSO (Environment for Modeling, Simulation and Optimization^48^ -https://www.enq.ufrgs.br/alsoc/download/index.php?dir=emso%2Fbin-win32). Detailed information about the equations employed for the biorefinery simulation can be found in Furlan^49^, Furlan *et al*.^27,30,50^, and Longati *et al*.^31^. The main data used for this analysis, together with the economic assumptions, were as described by Longati *et al*.^31^, with the exception of the solids mass fraction in the pretreatment, which was fixed at 10% (w/w), and the hydrolysis reaction time, which was set at 24 h. After the saccharification step, the soybean protein, together with the remaining lignocellulosic material, was sent to the boilers and was used to generate electricity (molar heating value = 1500 kJ/mol). Soybean protein was added to the process before the hydrolysis reactor, with heating to 50 °C, in order to avoid energy transfer problems in the hydrolysis reactor. Since the models and process configuration used in the 1G-2G sugarcane ethanol biorefinery did not change, the local sensitivity analysis performed by Longati *et al*.^31^ was used to select the most important process variables that greatly impact the economic performance of the process. The retro-techno-economic analysis (RTEA)^30^ was used to determine and evaluate the performance targets required to make the use of soybean protein, as a hydrolysis additive, economically feasible for the biorefinery. The economic metric target specified here was a null net present value (NPV = 0), for a pre-specified IRR of 11%. Analysis was made of the effects of the hydrolysis variables: solids loading, enzyme dosage, and soybean protein concentration and price.

The RTEA technique employed in this work differs from the conventional techno-economic analysis, in which the operational conditions must be specified, the simulation problem is solved and then the process variables are used to determine the economic performance. This conventional techno-economic analysis becomes intrinsically iterative if the objective is to determine which operational condition makes the process economically feasible. RTEA turn this problem upside-down. Instead of iteratively looking for a process configuration that meets a minimum economic performance (such as NPV = 0), the specification of NPV = 0 is imposed in the simulation (using a pre-defined IRR), as another equation that needs to be satisfied for the solver to converge. Hence, to keep the system well-posed (zero degrees of freedom), a process specification must be freed. Once the equations system is solved, the response is the value that this process variable should have to reach the economic specification (NPV = 0).

The resulting contour line is called isoeconomic, and it provides the value of a selected metric after NPV was set equal to 0, using a pre-defined minimum IRR. Thus, all points on that isoeconomic, which limits the economically feasible region, has NPV = 0, and the corresponding value of the metric under analysis is calculated when the overall process is simulated. For example, take the enzyme loads on the isoeconomic shown in Fig. 5c. They were not defined iteratively; instead of simulating the biorefinery with a specified load of enzyme and then calculating the value of NPV, the equation NPV = 0 replaced the specification of this metric within the set of equations that model the overall process. This methodology is conceived for equation-oriented process simulators, where all equations are solved simultaneously (in the case of the biorefinery herein studied, 27379 equations). Of course, in order to check whether the feasible region is above or below this curve, a local sensitivity analysis, based on derivatives, could be performed on any point of the isoeconomic curve. The signal of the derivative would answer this question.

In summary, the RTEA can be structured into four steps: (1) Construction of a base case; (2) Incorporation of TEA analysis into the process simulation; 3) Selection of key variables through sensitivity analysis; and (4) Delimitation of the feasible space. More detailed information about the technique can be found in Furlan *et al*.^30^.
